# Association between developmental dental anomalies, early childhood caries and oral hygiene status of 3–5-year-old children in Ile-Ife, Nigeria

**DOI:** 10.1186/s12903-019-0991-2

**Published:** 2019-12-31

**Authors:** Morenike Oluwatoyin Folayan, Michael Alade, Abiola Adeniyi, Maha El Tantawi, Tracy L. Finlayson

**Affiliations:** 1 Obafemi Awolowo University, Ile-Ife, Nigeria; 2 Obafemi Awolowo University Teaching Hospitals’ Complex, Ile-Ife, Nigeria; 3 Lagos State University, Lagos, Nigeria; 4 Alexandria University, Alexandria, Egypt; 5 San Diego State University, San Diego, CA USA

**Keywords:** Early childhood caries, Developmental anomalies, Nigeria, Preschool children, Hypomineralized second primary molar, Enamel hypoplasia, Oral hygiene

## Abstract

**Background:**

To determine the association between developmental dental anomalies (DDA), early childhood caries (ECC) and oral hygiene status of 3–5-year-old children resident in Ile-Ife, Nigeria.

**Methods:**

This was a cross-sectional study. We analyzed data for 3–5-year-olds extracted from the dataset of a household survey collected to determine the association between ECC and maternal psychosocial wellbeing in children 0–5-year-old. The outcome variables for the study were ECC and poor oral hygiene. The explanatory variable was the presence of developmental dental anomalies (supernumerary, supplemental, mesiodens, hypodontia, macrodontia, microdontia, peg-shaped lateral, dens evaginatus, dens invaginatus, talons cusp, fusion/germination, hypoplasia, hypomineralized second molar, fluorosis, amelogenesis imperfecta). The prevalence of each anomaly was determined. Poisson regression analysis was conducted to determine the association between presence of developmental dental anomalies, ECC and oral hygiene status. The model was adjusted for sex, age and socioeconomic status.

**Results:**

Of the 918 children examined, 75 (8.2%) had developmental dental anomalies, 43 (4.7%) had ECC, and 38 (4.1%) had poor oral hygiene. The most prevalent developmental dental anomalies was enamel hypoplasia (3.9%). Of the 43 children with ECC, 6 (14.0%) had enamel hypoplasia and 3 (7.6%) had hypomineralized second primary molar. There was a significant association between ECC and enamel hypoplasia (*p* < 0.001) and a borderline association between ECC and hypomineralized second primary molars (*p* = 0.05). The proportion of children with poor oral hygiene (PR: 2.03; 95% CI: 0.91–4.56; *p* = 0.09) and ECC (PR: 2.02; 95% CI: 0.92–4.46; *p* = 0.08) who had developmental dental anomalies was twice that of children with good oral hygiene and without ECC respectively, although the differences did not reach statistical significance.

**Conclusions:**

Enamel hypoplasia and hypomineralized second primary molars are developmental dental anomalies associated with ECC. developmental dental anomalies also increases the probability of having poor oral hygiene in the population studied.

## Background

Dental hard-tissue anomalies consist of anomalies of tooth shape, tooth size, tooth structure, tooth number, and tooth position [1]. The prevalence of these lesions can differ significantly across and among populations. Population-specific data on developmental dental anomalies (DDA) are of epidemiological and anthropological importance because the inter-relatedness of many clinical dental features – normal and pathological – reflects the pleiotropic effects of genes during tooth development. The features provide insight into population dynamics and evolution and are helpful for the fields of dental paleopathology [2], clinical pediatric dentistry, and orthodontic practice as well as for understanding the evolution of human dentition [3].

Only 90 (46.6%) of the 193 United Nations countries have English-language publications on the epidemiology of DDA, despite several years of research on the subject. Data were least available for Africa, [3] and few studies had been conducted in Nigeria [4–6]. This study provides data on clinically diagnosed DDA in the primary dentition of young children in a suburban population in Nigeria. The aim of the study was to determine the association between ECC, oral hygiene status and DDA.

## Methods

This is a cross-sectional study. Data on DDA were collected as part of a household survey to determine the prevalence of early childhood caries (ECC) and associated maternal psychosocial risk factors. The study participants were 1549 children aged 6 to 71 months resident in Ife Central Local Government Area of Osun, State, Ile-Ife, a semi-urban community. The survey was conducted between December 2018 and January 2019.

### Sample size

The minimum sample size for the study was calculated using the formula for cross-sectional studies in populations greater than 10,000 [7]. The variables for determining the sample size included the prevalence of ECC prevalence which was 6.6% [8], the margin of error was 5%, and the confidence level was 95%. The minimum sample size required for the study was 1439 mother-child dyads.

### Sampling and recruitment procedure

A multi-stage sampling technique was used for the study. The first stage consisted of selecting 70 of the 700 enumeration areas in the local government by a simple random, balloting method. This percentage of the population is considered representative for a household survey [9]. The second stage consisted of selecting eligible households within the enumeration sites of the survey. At each of the enumeration sites, every other household on each street was eligible for recruitment of a mother-child dyad. The third stage consisted of selecting respondents for an interview and clinical examination.

Only one child and mother dyad in each household was eligible for participation in the study. Only children who were below the age of 6 years, who were living with a caregiver, who were present at the time of the survey and for whom parental consent for study participation was obtained, were recruited for the study. Children who had chronic medical conditions that required prolonged use of sweetened medications and those with medical conditions that increased their risk for caries were excluded from the study. Where there was more than one child eligible for study participation, the study participant was identified by balloting. The other child (ren) was/were offered the opportunity to have a clinical examination. All participants were informed about the screening outcome and referred for treatment when oral diseases were identified. Treatment for ECC was offered free to study participants.

Whenever a household declined (19 households declined) to participate, the next eligible household was substituted. The number of participants to be recruited from each enumeration site was determined by proportioning the study’s sample size per the population of the enumeration site. Ethical approval for the study was obtained from the Obafemi Awolowo University Teaching Hospitals Complex Health Research Ethics Committee (NHREC/27/01/2009a & IRB/EC/0004553).

### Data collection

Data obtained included the children’s socio-demographic characteristics (age, sex, and socioeconomic status). The definition of socioeconomic status was based on a multiple-item index [10] that had been used in prior studies in Nigeria [10, 11]. The composite score was based on maternal occupation and paternal education. Enrolled children were classified according to one of five socio-economic classes: class I, upper class; class II, upper-middle class; class III, middle class; class IV, lower middle class; and class V, lower class. The classes were then categorized as high (Classes I and II), middle (Class III), and low (Classes IV and V).

The oral examinations were conducted by five calibrated dentists. Intra-examiner agreement for each of the dentists was calculated by use of the paired sample correlation, whereas the inter-examiner agreement (between the dentists and the trainer) was calculated by use of Cohen’s kappa coefficient. The intra- and inter-examiner reliability tests were all greater than 0.80, which is considered an acceps level of agreement. Children were examined under natural light, either sitting on a chair or on their mother’s lap.

The teeth were examined wet for the oral hygiene status assessment; thereafter, debris was removed with gauze before examination for the presence of ECC and DDA. Oral hygiene was assessed by use of the index of Greene and Vermillion [12]. The index teeth and surfaces examined were the facial and lingual surfaces of teeth number 51, 55, 65, 71, 75, and 85. The debris and calculus scores were recorded, added, and divided by the number of surfaces examined to get the OHI-S score. Oral hygiene was scored good when the scores ranged from 0.0 to 1.2; fair when the scores ranged from 1.3 to 3.0; or poor when the scores was 3.1 and above. For children who did not have the index teeth, all the teeth present were scored, and their average determined before being classified.

ECC was determined using the decayed-missing-filled teeth (dmft) index as recommended by the World Health Organization [13]. The dmft score was obtained by adding the d, m and f scores for each child. ECC was considered present when the dmft score was > 0 and absent when the dmft was 0.

The presence or absence of any DDA listed in Table 1 was determined according to the criteria of Temilola et al. [5] and Folayan et al. [14]. Also, hypomineralized primary second molar was defined as demarcated white, yellow or brown opacities that were greater than or equal to 2 mm in diameter, present on any of the surfaces of the primary second molar [15, 16]. Fluorosis assessed as presence or absence of tooth mottling [17]. A diagnosis of amelogenesis imperfecta was made when there was enamel hypoplasia and/or hypomaturation or hypocalcification randomly affecting multiple teeth in no depictable chronological order [18].

**Table 1:** Prevalence of clinically diagnosed developmental dental hard tissue anomalies by sex and socioeconomic status in 3–5 year old children in Ile-Ife (*N* = 918)

Variables		Sex					Socio-economic status							TOTAL *N* = 918	
		Male *n* = 495		Female *n* = 423		*P* value	High *n* = 291		Middle *n* = 402		Low *n* = 225		*P* value		
		Number	%	Number	%		Number	%	Number	%	Number	%		Number	%
Supernumerary	Absent	494	99.8	420	99.3	0.34	290	99.7	400	99.5	224	99.5	1.00	914	99.6
	Present	1	0.2	3	0.7		1	0.3	2	0.5	1	0.5		4	0.4
Supplemental	Absent	493	99.4	420	99.3	1.00	290	99.7	400	99.5	222	98.6	0.39	912	99.3
	Present	3	0.6	3	0.7		1	0.3	2	0.5	3	1.4		6	0.7
Mesiodens	Absent	492	99.6	418	98.8	0.26	289	99.3	400	99.5	222	98.6	0.55	911	99.2
	Present	2	0.4	5	1.2		2	0.7	2	0.5	3	1.4		7	0.8
Hypodontia	Absent	493	99.4	418	98.8	0.48	288	99.0	399	99.2	223	99.1	0.91	910	99.1
	Present	3	0.6	5	1.2		3	1.0	3	0.8	2	0.9		8	0.9
Macrodontia	Absent	494	99.8	419	99.0	1.90	290	99.6	399	99.2	224	99.5	0.86	913	99.4
	Present	1	0.2	4	1.0		1	0.4	3	0.8	1	0.5		5	0.6
Microdontia	Absent	493	99.6	419	99.0	0.42	289	99.3	400	99.5	223	99.1	0.87	912	99.3
	Present	2	0.4	4	1.0		2	0.7	2	0.5	2	0.9		6	0.7
peg-shaped lateral	Absent	491	99.2	416	98.3	0.36	288	98.9	398	99.0	221	98.2	0.69	907	98.8
	Present	4	0.8	7	1.7		3	1.1	4	1.0	4	1.8		11	1.2
Dens evaginatus	Absent	494	99.8	420	99.3	0.34	290	99.7	400	99.5	224	99.5	1.00	914	99.6
	Present	1	0.2	3	0.7		1	0.3	2	0.5	1	0.5		4	0.4
Dens invaginatus	Absent	493	99.6	420	99.3	0.67	290	99.7	400	99.5	223	99.1	0.73	913	99.4
	Present	2	0.4	3	0.7		1	0.3	2	0.5	2	0.9		5	0.6
Talons cusp	Absent	492	99.4	420	99.3	1.00	290	99.7	400	99.5	222	98.6	0.50	912	99.3
	Present	3	0.6	3	0.7		1	0.3	2	0.5	3	1.4		6	0.7
Fusion/gemination	Absent	494	99.8	420	99.3	0.34	290	99.7	401	99.7	223	99.1	0.47	914	99.6
	Present	1	0.2	3	0.7		1	0.3	1	0.3	2	0.9		4	0.4
Hypoplasia	Absent	479	96.7	404	95.4	0.39	281	96.5	387	96.2	215	95.5	0.86	883	96.1
	Present	16	3.3	19	4.6		10	3.5	15	3.8	10	4.5		35	3.9
Hypomineralised Second molar	Absent	485	97.9	415	98.1	1.00	285	97.9	395	98.2	220	97.7	0.87	900	98.0
	Present	10	2.1	8	1.9		6	2.1	7	1.8	5	2.3		18	2.0
Fluorosis	Absent	491	99.2	421	99.5	0.69	288	98.9	400	99.5	224	99.5	0.68	912	99.3
	Present	4	0.8	2	0.5		3	1.1	2	0.5	1	0.5		6	0.7
Amelogenesis imperfecta	Absent	491	99.2	420	99.3	1.00	290	99.7	399	99.2	222	98.6	0.44	911	99.2
	Present	4	0.8	3	0.7		1	0.3	3	0.8	3	1.4		7	0.8

### Data analysis

Data for this study were derived from the 918 young children aged 3–5 years old who had all their primary teeth erupted. Tests of association were conducted using chi-square or fisher test where appropriate. Descriptive analysis was conducted to determine the prevalence of each lesion. Multivariable logistic regression was conducted by use of the Poisson regression analysis to determine if the presence of a DDA, ECC and oral hygiene was associated. The estimated coefficients, expressed as prevalence ratios (PRs) and their 95% confidence intervals, were calculated. We chose to use the Poisson regression analysis because it reduces the risk for overestimation of the prevalence ratio especially when an adjusted estimate needs to be made. The analysis also used a robust variance estimator to allow for direct estimation of the PRs. Statistical analyses were conducted with Intercooled STATA (release 15) for windows. Statistical significance was inferred at *p* < 0.05.

## Results

Seventy-five (8.2%) children had DDA. Fifty-eight (77.3%) of the children with DDA had one anomaly, 12 (15.4%) had two anomalies, and five (6.7%) had three or more anomalies. The prevalence of DDA by sex and socioeconomic status are listed in Table 1; the most prevalent lesions were enamel hypoplasia (3.9%), hypomineralized primary second molar (2.0%), and peg-shaped laterals (1.2%). There were no significant differences in the prevalence of any of the DDA by sex and socioeconomic status.

Table 2 shows the prevalence of DDA by ECC and oral hygiene status. ECC was only identified in children with enamel hypoplasia and hypomineralized primary second molar. Six (14.0%) and three (7.0%) children with ECC had enamel hypoplasia and hypomineralized primary second molar, respectively. There was a statistically significant association between ECC, enamel hypoplasia (x^2^ = 12.24; *p* < 0.001) and hypomineralized second molar (Fisher’s exact test: *p* = 0.05): a higher proportion of children with ECC had enamel hypoplasia (3.4% vs 14.0%) and hypomineralized second molar (1.8% vs 7.0%) when compared with children without ECC.

**Table 2:** Prevalence of clinically diagnosed developmental dental hard tissue anomalies by caries and oral hygiene status in preschool children in Ile-Ife (N = 918)

Variables		Early childhood caries					Oral hygiene status							TOTAL *N* = 918	
		Absent *N* = 875		Present *n* = 43		*p*- value	Good *n* = 613		Fair *n* = 267		Poor *n* = 38		*p*- value		
		Number	%	Number	%		Number	%	Number	%	Number	%		Number	%
Supernumerary	Absent	871	99.5	43	100.0	1.00	610	99.5	267	100.0	37	97.4	0.10	897	99.6
	Present	4	0.5	0	0.0		3	0.5	0	0.0	1	2.6		4	0.4
Supplemental	Absent	869	99.3	43	100.0	1.00	609	99.3	266	99.6	37	97.4	0.28	896	99.3
	Present	6	0.7	0	0.0		4	0.7	1	0.4	1	2.6		6	0.7
Mesiodens	Absent	868	99.2	43	100.0	1.00	610	99.5	265	99.2	36	94.7	0.03	889	99.2
	Present	7	0.8	0	0.0		3	0.5	2	0.8	2	5.3		7	0.8
Hypodontia	Absent	867	99.1	43	100.0	1.00	608	99.2	267	100.0	35	92.1	0.03	896	99.1
	Present	8	0.9	0	0.0		5	0.8	0	0.0	3	7.9		8	0.9
Macrodontia	Absent	870	99.4	43	100.0	1.00	609	99.3	267	100.0	37	97.4	0.007	898	99.4
	Present	5	0.6	0	0.0		4	0.7	0	0.0	1	2.6		5	0.6
Microdontia	Absent	869	99.3	43	100.0	1.00	608	99.2	267	100.0	37	97.4	0.10	890	99.3
	Present	6	0.7	0	0.0		5	0.8	0	0.0	1	2.6		6	0.7
peg-shaped lateral	Absent	864	98.7	43	100.0	1.00	607	99.0	263	98.5	37	97.4	0.34	888	98.8
	Present	11	1.3	0	0.0		6	1.0	4	1.5	1	2.6		11	1.2
Dens evaginatus	Absent	871	99.5	43	100.0	1.00	610	99.5	267	100.0	37	97.4	0.10	898	99.6
	Present	4	0.5	0	0.0		3	0.5	0	0.0	1	2.6		4	0.4
Dens invaginatus	Absent	870	99.4	43	100.0	1.00	609	99.3	267	100.0	37	97.4	0.10	896	99.4
	Present	5	0.6	0	0.0		4	0.7	0	0.0	1	2.6		5	0.6
Talons cusp	Absent	869	99.3	43	100.0	1.00	608	99.2	267	100.0	37	97.4	0.10	893	99.3
	Present	6	0.7	0	0.0		5	0.8	0	0.0	1	2.6		6	0.7
Fusion/gemination	Absent	871	99.5	43	100.0	1.00	609	99.3	267	100.0	38	100.0	0.43	893	99.6
	Present	4	0.5	0	0.0		4	0.7	0	0.0	0	0.0		4	0.4
Hypoplasia	Absent	846	96.6	37	86.0	< 0.001	585	95.3	262	98.1	36	94.7	0.10	865	96.1
	Present	29	3.4	6	14.0		28	4.7	5	1.9	2	5.3		35	3.9
Hypomineralised Second molar	Absent	860	98.2	40	93.0	0.05	599	97.7	264	98.9	37	97.4	0.37	879	98.0
	Present	15	1.8	3	7.0		14	2.3	3	1.1	1	2.6		18	2.0
Fluorosis	Absent	869	99.3	43	100.0	1.00	609	99.3	265	99.2	38	100.0	1.00	886	99.3
	Present	6	0.7	0	0.0		4	0.7	2	0.8	0	0.0		6	0.7
Amelogenesis imperfecta	Absent	868	99.2	43	100.0	1.00	607	99.0	267	100.0	37	97.4	0.10	886	99.2
	Present	7	0.8	0	0.0		6	1.0	0	0.0	1	2.6		7	0.8

Also, there were 267 (29.1%) children with fair oral hygiene and 38 (4.1%) with poor oral hygiene. More children with microdontia (2.6% vs 0.7% vs 0.0%; *p* = 0.007), hypodontia (7.9% vs 0.8% vs 0.0%, *p* = 0.03), and mesiodens (5.3% vs 0.5% vs 0.8%, p = 0.03) had poor oral hygiene compared to those with good and fair oral hygiene respectively.

Table 3 shows the variables associated with the presence of DDA in the primary dentition. The proportion of children with DDA who had poor oral hygiene was twice that of children who had good oral hygiene (PR: 2.03; 95% CI: 0.91–4.56; *p* = 0.09), and the proportion of children with DDA who had ECC was twice that of children without ECC (PR: 2.02; 95% CI: 0.92–4.46; *p* = 0.08), but these differences were not statistically significant.

**Table 3:** Factors associated with presence of developmental dental tissue anomalies in the primary dentition (n-918)

Variables	Prevalence Ratio	Standard Error	Z	P value	[95% Confidence Interval]	
Sex						
Male	1.00	–	–	–	–	–
Female	1.14	0.28	0.53	0.59	0.70	1.84
Socio-economic status						
High	1.00	–	–	–	–	–
Middle	1.05	0.33	0.16	0.87	0.57	1.95
Low	1.81	0.57	1.86	0.06	0.97	3.37
Oral hygiene status						
Good	1.00	–	–	–	–	–
Fair	0.68	0.21	−1.25	0.21	0.37	1.24
Poor	2.03	0.84	1.72	0.09	0.91	4.56
Early childhood caries						
Absent	1.00	–	–	–	–	–
Present	2.02	0.82	1.74	0.08	0.92	4.46
Constant	0.06	0.02	- 10.35	< 0.001	0.03	0.10

## Discussion

This study adds new information to the existing evidence on the prevalence of DDA in a low- middle-income suburban population in Nigeria. It is the first study on the prevalence of DDA among preschool-aged children in Nigeria, although a prior study reported the prevalence of DDA lesions in primary teeth of children aged 6–12 years old [6]. The present study is the first to provide population-level estimates of the prevalence of hypomineralized primary second molar, fluorosis, and amelogenesis imperfecta in children aged 3–5 years old in Nigeria. It is also the first to report an association between ECC, enamel hypoplasia and hypomineralized primary second molar in Nigeria. Although several studies have shown that enamel hypoplasia was the most prevalent DDA in Nigeria [19], and that there was an association between caries and hypomineralized primary second molar [20], none highlighted a relationship between caries and enamel hypoplasia. Prior to this study, none have explored the association between ECC, fluorosis, and amelogenesis imperfecta.

One strength of this study is the method of data collection: the use of a household survey from enumeration sites marked for population surveys in Nigeria. This method increased the appropriate representation of the study population through the study-sampling approach, and our findings are more generalizable to 3–5-year old children in this region. In addition, the household survey is an appropriate recruitment strategy for reaching preschool-aged children who would not be recruited through school surveys.

The study showed that the prevalence of DDA in the primary dentition of preschoolers was lower than the 11.3 and 16.1% reported in children with mixed dentition in earlier studies, [19] but higher than the 2.2% reported in the permanent dentition of study participants in Nigeria [6]. The prevalence of DDA reported in this study is higher than 1.8 to 2.5% that was reported for preschool children in Brazil [21].

The prevalence of enamel hypoplasia in this population is lower than the 15.5% reported in preschool children in Brazil [22] and 7.6% reported in 6–36-month old children in Tanzania [23]. Also, a study conducted on the prevalence of enamel hypoplasia in 1- and 4-year-old children in Tanzania revealed that the prevalence of enamel hypoplasia ranged from 2.7 to 37.2%, with prevalence higher in regions with lower fluoride content in drinking water [24]. Though the fluoride level of natural water in the study region (le-Ife) is 1.11–1.50 ppm [25], like the fluoride level of the Dodoma region of Tanzania (1.5 ppm), the prevalence of enamel hypoplasia is less than that of the Dodoma region (5.7%). The differences in the prevalence of enamel hypoplasia within and across countries may not be related to access to fluoride in drinking water, but this issue should be explored further.

We found no significant association between the presence of DDA and the prevalence of ECC and poor oral hygiene. However, we found that the proportion of children with ECC and poor oral hygiene who had a DDA was twice that of those who had no ECC and those who had good oral hygiene. The presence of a DDA increases the risk for plaque retention [6], which is a known risk factor for ECC in Nigeria [26]. A prior study had shown that DDA increased the risk for poor oral hygiene in the permanent dentition [6]. Although our study found no statistically significant association between DDA, ECC and oral hygiene status, the higher prevalence level of ECC and poor oral hygiene with a DDA may imply that children 3–5 years old with DDA should be treated as children with high risk for ECC and poor oral hygiene.

The study had a few limitations one of which is that this was a cross-sectional study, so no causal association between DDA, ECC and oral hygiene status can be inferred. The study was not representative of all preschool children in Nigeria; data were collected from only one of the 774 local government areas in the country. For the study population however, the finding from this study can be representative of the prevalence of DDA for the study population as the sample size was powered enough to address the study objective using a prevalence of 26.6% determined in a prior study conducted in the study population [5]. The prevalence of the DDA was however, an underestimation of the true prevalence of DDA in the study population as intra-bony lesions like supernumerary teeth, were not determined using radiographs. Despite these limitations, this study generated new and important information for the study population that can be useful for public health response.

## Conclusion

We found ECC was associated with enamel hypoplasia and hypomineralized second primary molars. Also, the probability of a preschool child having ECC and having poor oral hygiene was increased in children with DDA though this finding did not reach statistical significance. In view of the known association between poor oral hygiene and ECC, screening for these DDA may be a strategic public health intervention to reduce the risk of children for ECC in the study population.

## Data Availability

Study-related data and materials and data collection tool are accessible on request from the lead author.

## Abbreviations

CI: Confidence Interval
DDA: Developmental Dental Anomalies
ECC: Early childhood caries
PR: Prevalence Ratio
